# Biomolecules and Natural Medicine Preparations: Analysis of New Sources of Bioactive Compounds from *Ribes* and *Rubus* spp. Buds

**DOI:** 10.3390/ph9010007

**Published:** 2016-02-05

**Authors:** Dario Donno, Maria Gabriella Mellano, Alessandro Kim Cerutti, Gabriele Loris Beccaro

**Affiliations:** Department of Agriculture, Forestry and Food Science, University of Torino, Largo Braccini 2, 10095 Grugliasco (TO), Italy; gabriella.mellano@unito.it (M.G.M.); alessandro.cerutti@unito.it (A.K.C.); gabriele.beccaro@unito.it (G.L.B.)

**Keywords:** biomarkers, *Ribes nigrum*, *Rubus* cultivated varieties, bioactivity, herbal preparations, phytochemical fingerprint, bud-extracts

## Abstract

It is well known that plants are important sources for the preparation of natural remedies as they contain many biologically active compounds. In particular, polyphenols, terpenic compounds, organic acids, and vitamins are the most widely occurring groups of phytochemicals. Some endemic species may be used for the production of herbal preparations containing phytochemicals with significant bioactivity, as antioxidant activity and anti-inflammatory capacities, and health benefits. Blackberry sprouts and blackcurrant buds are known to contain appreciable levels of bioactive compounds, including flavonols, phenolic acids, monoterpenes, vitamin C, and catechins, with several clinical effects. The aim of this research was to perform an analytical study of blackcurrant and blackberry bud-preparations, in order to identify and quantify the main biomarkers, obtaining a specific phytochemical fingerprint to evaluate the single botanical class contribution to total phytocomplex and relative bioactivity, using a High Performance Liquid Chromatograph−Diode Array Detector; the same analyses were performed both on the University laboratory and commercial preparations. Different chromatographic methods were used to determine concentrations of biomolecules in the preparations, allowing for quantification of statistically significant differences in their bioactive compound content both in the case of *Ribes nigrum* and *Rubus* cultivated varieties at different harvest stages. In blackcurrant bud-extracts the most important class was organic acids (50.98%) followed by monoterpenes (14.05%), while in blackberry preparations the main bioactive classes were catechins (50.06%) and organic acids (27.34%). Chemical, pharmaceutical and agronomic-environmental knowledge could be important for obtaining label certifications for the valorization of specific genotypes, with high clinical and pharmaceutical value: this study allowed to develop an effective tool for the natural preparation quality control and bioactivity evaluation through the chemical fingerprinting of bud preparations.

## 1. Introduction

Plants are important sources for the preparation of natural remedies, food additives, and other ingredients, as they contain many biologically active compounds as polyphenols, vitamins (A, B group, C, E), terpenes, organic acids, and other very important phytochemicals [1,2]. For this reason, plant material and herbal preparations have been widely used for hundreds of years all over the world [3]: they have provided a complete storehouse of remedies to cure acute and chronic diseases. Berry species have been demonstrated to exhibit a broad spectrum of benefits; in particular, blackberry (*Rubus* cultivated varieties) sprouts and blackcurrant (*Ribes nigrum* L.) buds are known to contain appreciable levels of vitamins, terpenic and phenolic compounds, including flavonols, phenolic acids and catechins [4,5]. The most important industrial product of blackcurrant is its fruits, however, the leaves and buds, due to their characteristic chemical composition and excellent flavor, have also found some applications as a raw material for the herbal and cosmetic industries. Many people use its buds as a medicinal preparation for its anti-inflammatory activity and against dermal diseases (eczema and psoriasis) [6,7]. On the other hand, blackberry sprouts have been used in traditional medicine for their medicinal antioxidant, anti-haemorrhoidal and anti-diarrhoeal activity properties [8,9].

Phytotherapy is the study of natural extracts used as health-promoting products for medical care [10]. The idea comes from the observation that certain plants, or parts thereof, taken as food, may have therapeutic effects. Every early civilization has used plants or parts of plants (buds, leaves, sprouts, flowers, fruits, seeds, bark, roots) as their main source of healthcare, and this holds true even today in many rural populations [11]. Moreover, there is also a greater tendency toward regular use of alternative therapies in the main European countries: 49% and 46% of the population in France and Germany, respectively, use them regularly, along with 35%, 31%, and 25% of the population in the United Kingdom, Belgium, and the Northern Europe countries, respectively [12]. Natural medicine has not been officially recognized in most countries [13], but increasing acceptance by consumers and medical professionals has pushed world demand for herbal extracts up 7.5% annually to US $1.95 billion in 2012 [14,15].

Gemmotherapy is the most recent of therapeutic techniques developed on the basis of the plant medical properties. It uses the properties of extracts obtained by the maceration in ethanol and glycerol of fresh meristematic plant tissues, mainly buds and sprouts, for medicinal purposes. The products are commercially known as bud-preparations. In herbal preparations, due to the large quantity of bioactive compounds, many of which act synergistically, there is a preference to attribute the pharmacological effect to the “phytocomplex” (a combination of different substances, both active principles and other plant components), rather than to any single active compound, as in the case of standard medicine [16].

In recent years phytotherapy has become a fully fledged medical discipline, as the knowledge gleaned from folk medicine has since been subjected to methodical scientific assessments in order to provide evidence of its efficacy [17]. However, the fast growth of the herbal products industry and the lack of corresponding regulations and legislations have caused the World Health Organization (WHO) and other regulatory bodies to be increasingly concerned with the safety and efficacy of herbal medicines [18,19]. In particular, research on bud-preparations, until now, has been only focused on their clinical effects and research on raw material origins, cultivation and quality is still lacking [20]. Instead, quality control of natural products is extremely important, as the effectiveness and quality of herbal medicines depend on the concentrations of their active ingredients [21]. Key factors that can affect the quality and quantity of these compounds include the plant genotype, pedoclimatic conditions, applied agronomic techniques and phenological stage in which the buds are harvested [22,23]. Moreover, the herbal preparation quality is also determined by the subsequent processing and storage procedures [24].

The lack of information on the intrinsic and extrinsic factors that determine the quality and effectiveness of bud-preparations indicates the need to extend research on this topic, however, due to the variability and complexity of bud-preparations, it is very difficult to control their product quality [25]. The key factors in achieving this objective are the determination of chemical composition and the standardization of herbal preparations. The definition of a chromatographic (High Performance Liquid Chromatography—HPLC) fingerprint allows for the qualitative and quantitative evaluation of phytocomplex components [26,27]. In particular, the best method of identifying preparations is by measuring the concentration of the main bioactive compounds, called “biomarkers” [28,29].

Given the above, the aim of this research was to perform an analytical study of blackcurrant and blackberry bud-preparations using simple, sensitive and reliable HPLC–diode array detector (DAD) methods in order to identify and quantify the main phytochemicals (biomarkers) and to be able to obtain a specific botanical fingerprint for the assessment of the single bioactive class contribution to total bud preparation phytochemical profile The influence of genotype and harvest stage on these bioactive substances in the bud-extracts was also analysed. The same analyses were performed both on laboratory preparations and on commercial preparations.

## 2. Results and Discussion

Recently, many screening studies of different plant materials have been performed in order to find naturally occurring antioxidant compounds for use in food or medicinal preparations as replacements for potentially harmful synthetic additives [30]. Phenolic acids, flavonols and catechins were often selected for quantitative studies [31,32]. In this case, the extracts of the analyzed species are recommended by physicians to be consumed as phytochemical supplements, and further information could be used to direct future research towards condition-specific beneficial properties associated with their therapeutic effects [33,34].

The chemical composition of secondary plant metabolites is highly dependent on several factors such as climatic conditions, harvesting time, and plant genotype [35,36,37]. The present study showed that bioactive compound concentration in bud-preparations can be properly defined and characterized on the basis of chemical, agricultural and environmental knowledge. Different genotypes presented different chemical composition, but it was also important to consider that the pedoclimatic conditions of sampling sites strongly influence the presence of these compounds, as comparing the results of commercial bud-preparations.

**Figure 1:**
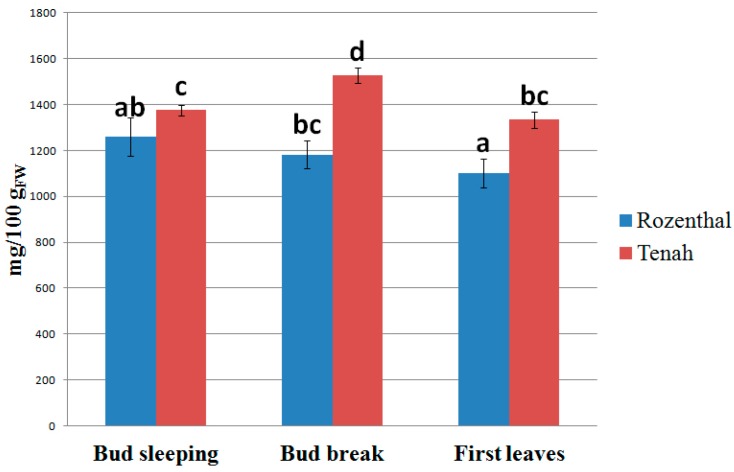
Effect of bud phenological stage on the bioactive compound content (TBCC) in final blackcurrant bud-preparations. Different letters for each sample indicate the significant differences at *p* < 0.05.

Blackcurrant bud-preparations have been identified as herbal products with a high health-value (Figure 1). The second phenological stage (bud break) was the best for the blackcurrant bud harvesting because it presented the highest values of bioactive compounds, followed by the first step (bud sleeping) and the third one (first leaves). In all the phenological stages, Tenah cultivar showed a greater phytochemical content (1527.70 mg/100 g_FW_, bud break) than Rozenthal cultivar (1181.11 mg/100 g_FW_, bud break).

**Figure 2:**
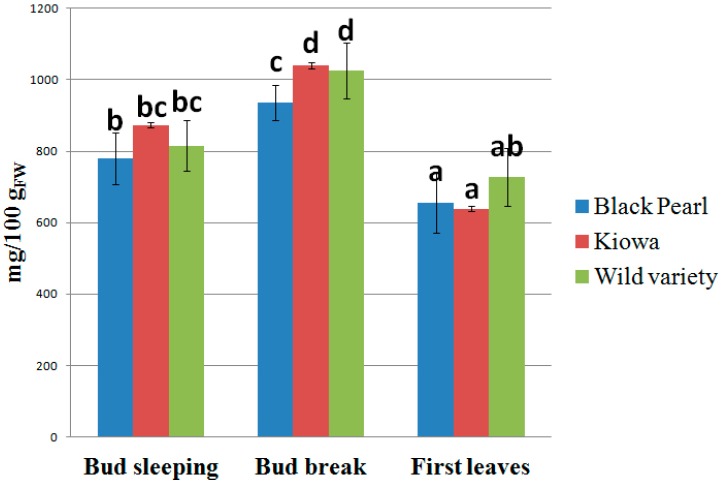
Effect of bud phenological stage on the bioactive compound content in blackberry final bud-preparations. Different letters for each sample indicate the significant differences at *p* < 0.05.

**Figure 3:**
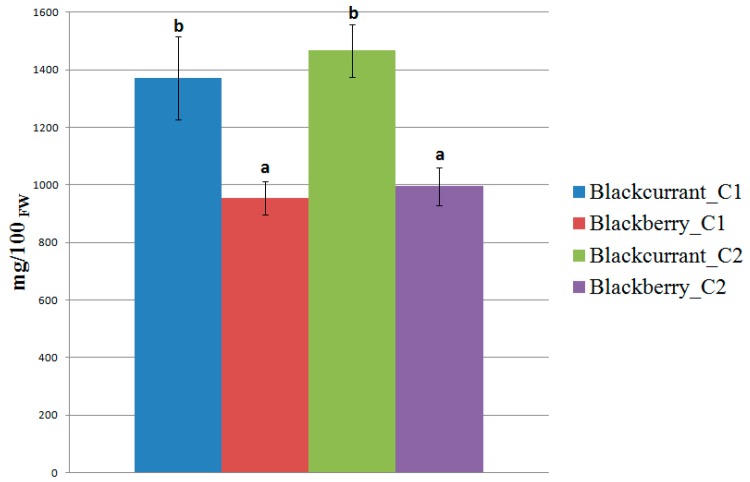
Total bioactive compound content in commercial bud-preparations. Different letters for each sample indicate the significant differences at *p* < 0.05.

The blackberry herbal preparations showed different chemical compositions with a high antioxidant compound content. Bud break was again the best phenological stage for bud harvesting, followed by the first step and third one (Figure 2). Kiowa cultivar (1039.78 mg/100 g_FW_, bud break) and wild variety (1026.73 mg/100 g_FW_, bud break) presented a greater total bioactive compound content (TBCC) than Black Pearl cultivar (935.98 mg/100 g_FW_, bud break). As reported in similar studies [38], the analysis carried out on commercial bud-products highlighted significant statistical differences between species (RC1 *vs*. RRC1 and RC2 *vs.* RRC2), but there were no differences between companies (RC1 *vs*. RC2 and RRC1 *vs.* RRC2) (Figure 3), confirming a production supply chain standardized according to the official Pharmacopoeia protocols.

**Figure 4:**
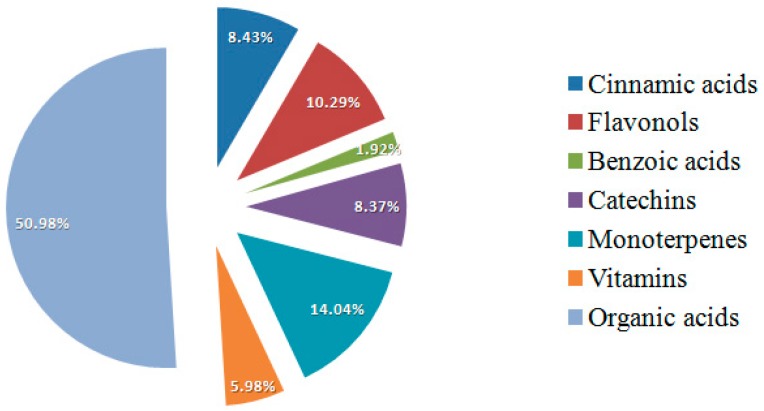
Contribution of each bioactive class to blackcurrant total phytocomplex. For the phytocomplex graphical representation, the second phenological stage was selected (bud break). Mean values of all the analyzed genotypes were considered.

**Figure 5:**
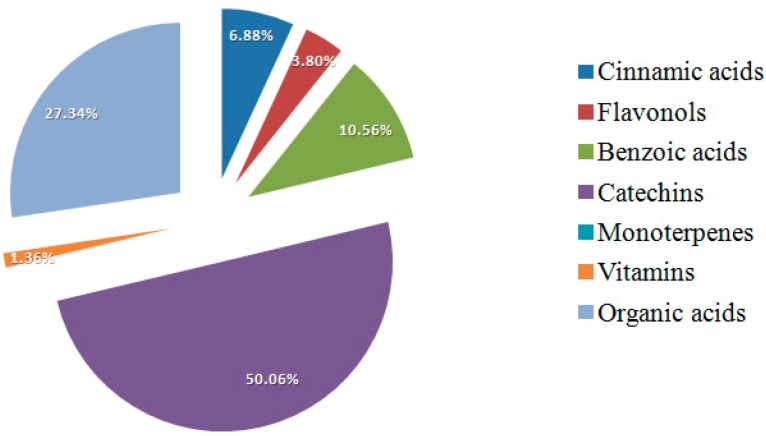
Contribution of each bioactive class to blackberry total phytocomplex. For the phytocomplex graphical representation, the second phenological stage was selected (bud break). Mean values of all the analyzed genotypes were considered.

**Figure 6:**
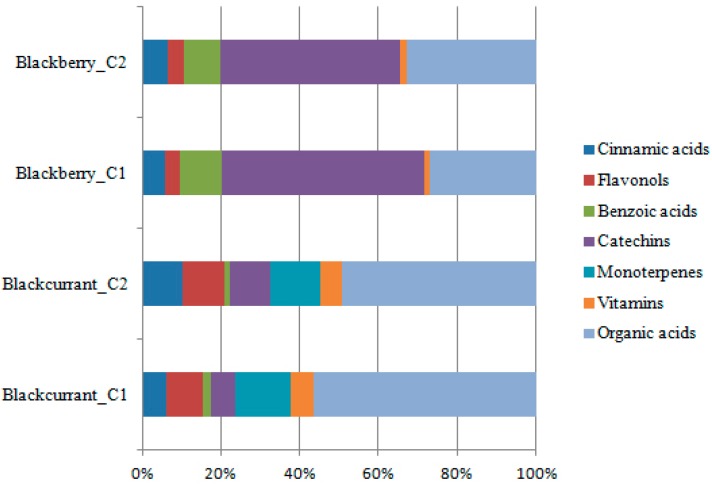
Contribution of each bioactive class to total phytocomplex in commercial bud-preparations.

Bud-preparation phytochemical fingerprints of the selected genotypes are reported. In total, 26 botanicals were evaluated by HPLC/DAD. By single bioactive compound profile, phytochemicals were grouped into single bioactive classes to evaluate the contribution of each class to total phytocomplex composition [39]. Fingerprint profiles showed the prevalence of different bioactive classes in chemical composition of all the analyzed preparations depending on genotype. In Figure 4, the *R. nigrum* bud-preparation phytocomplex (mean values were considered) showed the prevalence of organic acids (50.98%) and polyphenols (29.39%), followed by monoterpenes (14.04%) and vitamins (5.98%). In *Rubus* cultivated varieties bud-extracts phytocomplex (Figure 5), the most important bioactive class was polyphenols (71.03%), followed by organic acids (27.34%) and vitamins (1.36%). Monoterpenes were not detected in blackberry preparations. Commercial preparations of the same species from different companies showed similar phytocomplexes, while the differences among species were confirmed according to the previous results obtained on lab preparations; moreover, the percentage ratio between bioactive class content (polyphenols, monoterpenes, organic acids and vitamins) and TBCC confirmed these results (Figure 6).

The obtained fingerprints, and relative phytocomplexes, were useful for authentication and quality control purposes, as shown in other studies [40,41]. Most of the research pointed out that the identified antioxidant compounds (polyphenols and vitamins) contribute significantly to the total phytocomplex of herbal preparations [31,42]: the present study confirmed these results, adding as well as the terpenic and organic compounds also significantly contributed to the bud-preparation phytocomplex, as anti-inflammatory and volatile constituents in herbal preparations.

In this study, HPLC-DAD methods were used for fingerprint analysis and component identification of blackcurrant and blackberry bud-preparations. Comparing with other analytical studies [5,43], the chromatographic conditions were optimized in order to obtain a fingerprint with good peak resolution and reasonable analysis time for the separation and quantification of different bioactive classes in plant material derived-products. These methods could be applied in routine quality control and standardization of bud-extracts, germplasm evaluation and selection of new cultivars with high content of biomolecules, and phytochemical fingerprinting of the plant material to be used in pharmaceutical investigations, in particular avoiding substitutions, changes or adulterations with other species or synthetic drugs (e.g., sildenafil, diazepam, captopril and amoxicillin), as shown in other studies [44,45].

This study only focused on bud-preparation chemical composition of two berryfruit species, in order to detect and quantify the most important biologically active classes and single compounds, but a further quantitative evaluation on the basis of their native structures with Nuclear Magnetic Resonance (NMR) or HPLC coupled to mass spectrometry is necessary.

## 3. Materials and Methods

### 3.1. Plant Material

University lab preparations and commercial preparations were evaluated. Samples of *Ribes nigrum* L. (buds) and *Rubus* cultivated varieties (sprouts) were collected in 2014, in three different phenological stages (bud sleeping, bud break, and first leaves), from two germplasm repositories in Turin Province (Italy), Grugliasco (*Rubus* cultivated varieties) and San Secondo di Pinerolo (*R. nigrum*). Different genotypes were sampled, in order to test the genotype effect on the final product chemical composition (blackcurrant: Rozenthal and Tenah; blackberry: Black Pearl, Kiowa and a wild variety). Buds and sprouts were used fresh to prepare herbal preparations; HPLC samples were analyzed after being stored for a few days at normal atmosphere (N.A.), at 4 °C and 95% relative humidity (R.H.). Commercial products from two different Italian herbal companies were also analyzed: the companies are located in San Gregorio di Catania (Catania Province, Company 1), and Predappio (Forlì-Cesena Province, Company 2). Table 1 shows the genotypes, the sampling times and sites of analyzed herbal preparations (University and commercial preparations).

**Table 1:** Genotype, sampling time, provenience and identification code of the analyzed bud-preparations.

*University bud-preparations*				
Species	Genotype	Year	Germplasm repository	Identification code
*Ribes nigrum* L.	Rozenthal	2014	San Secondo di Pinerolo, Torino, Italy	RR
	Tenah			RT
*Rubus ulmifolius* Schott	Black Pearl	2014	Grugliasco, Torino, Italy	RRBP
	Kiowa			RRK
	Wild variety			RRW
***Commercial bud-preparations***				
**Species**	**Company**	**Year**	**Germplasm repository**	**Identification code**
*Ribes nigrum* L.	Company 1	2013	San Gregorio di Catania, Catania, Italy	RC1
	Company 2		Predappio, Forlì-Cesena, Italy	RC2
*Rubus ulmifolius* Schott	Company 1	2013	San Gregorio di Catania, Catania, Italy	RRC1
	Company 2		Predappio, Forlì-Cesena, Italy	RRC2

### 3.2. Solvents and Chemicals

Ethanol, hydrochloric acid, formic acid and all the organic acid standards were purchased from Fluka Biochemika (Buchs, Switzerland). Analytic HPLC grade acetonitrile, methanol, glycerol, all the polyphenolic and terpenic standards, potassium dihydrogen phosphate, 1,2-phenylenediamine dihydrochloride (OPDA) and phosphoric acid were purchased from Sigma Aldrich (Saint Louis, MO, USA). Milli-Q ultrapure water was produced by using a Sartorius Stedium Biotech mod. Arium apparatus (Sartorius, Goettingen, Germany). Cetyltrimethylammonium bromide (cetrimide), ascorbic and dehydroascorbic acids were purchased from Extrasynthése (Genay, France).

### 3.3. Sample Preparation Protocols

The extraction solution was prepared based on the protocol of bud-preparations detailed in the monograph “Homeopathic preparations”, quoted in the French Pharmacopoeia, 8th edition, 1965 [46]. The bud mother solutions were prepared using one part of the fresh material (calculated as dried weight) in 20 parts of glycerol-ethanol solution (1:1 ratio). Bioactive molecules were extracted through a cold maceration process for 21 days, in a solution of ethanol (95%) and glycerol, followed by a first filtration (Whatman Filter Paper, Hardened Ashless Circles, 185 mm Ø), a manual pressing and, after two days of decanting, a second filtration (Whatman Filter Paper, Hardened Ashless Circles, 185 mm Ø). Macerated preparations were filtered with circular pre-injection filters (0.45 µm, polytetrafluoroethylene membrane, PTFE) and then stored for a few days at N.A., 4 °C and 95% R.H until analysis. All samples were analyzed as such without dilution. For vitamin C analysis, 250 µL of OPDA solution (18.8 mmol/L) was added to 750 µL of extracted samples for dehydroascorbic acid derivatization into the fluorophore 3-(1,2-dihydroxyethyl)-furo(3,4-b)quinoxalina-1-one (DFQ). After 37 min in the dark the samples were analyzed with a High Performance Liquid Chromatograph (HPLC) coupled to a diode array detector (DAD) [10].

### 3.4. Apparatus and Chromatographic Conditions

An Agilent 1200 High Performance Liquid Chromatograph, equipped with a G1311A quaternary pump, a manual injection valve, and a 20 μL sample loop, coupled to an Agilent GI315D UV-Vis diode array detector (Agilent Technologies, Santa Clara, CA, USA), was used for the analysis. Five different chromatographic methods were used to analyze the samples, two for polyphenols and one for monoterpenes, organic acids, and vitamins, respectively. In all of the used methods, bioactive compound separation was achieved on a KINETEX–C18 column (4.6 × 150 mm, 5 μm, Phenomenex, Torrance, CA, USA). Different mobile phases were used for a specific bioactive compound identification and UV spectra were recorded at 330 nm (A); 280 nm (B); 210, 220, 235, and 250 (C); 214 nm (D); 261, and 348 nm (E). The chromatographic conditions of each method were reported in Table 2.

### 3.5. Identification and Quantification of Bioactive Compounds

All the single compounds were identified in samples by comparison and combination of their retention times and UV spectra with those of authentic standards in the same chromatographic conditions. The external standard method was used for quantitative determinations. Twenty μL aliquots of each standard solution were used for HPLC analysis and injections were performed in triplicate for each concentration level. For reference compounds, the limit of detection (LOD) and the limit of quantification (LOQ) were experimentally determined by HPLC analysis of serial dilutions of a standard solution to reach a signal-to-noise (S/N) ratio of 3 and 10, respectively. The main analytical method validation data are summarized in Table 3.

All samples were analyzed in triplicate, and standard deviations are given in order to assess the repeatability of the used methods. Accuracy was checked using the recovery test by spiking samples with a solution containing each bioactive compound (10 mg·mL^−1^) to reach 100% of the test concentration.

**Table 2:** Chromatographic conditions of each used method [10].

Method	Compounds of interest	Stationary phase	Mobile phase	Flow *(mL min^−1^)*	Time of analysis *(min)*	Gradient	Wavelenght *(nm)*
A	cinnamic acids, flavonols	KINETEX–C18 column (4.6×150 mm, 5 μm)	A: 10 mM KH_2_PO_4_/H_3_PO_4_, pH=2.8;	1.5	20+2 (CT)	Yes	330
			B: CH_3_CN				
B	benzoic acids, catechins	KINETEX–C18 column (4.6×150 mm, 5 μm)	A: H_2_O/CH_3_OH/HCOOH (5:95:0.1 v/v/v), pH=2.5;	0.6	23+2 (CT)	Yes	280
			B: CH_3_OH/HCOOH (100:0.1 v/v)				
C	monoterpenes	KINETEX–C18 column (4.6×150 mm, 5 μm)	A: H_2_O;	1.0	17+3 (CT)	Yes	210, 220,
			B: CH_3_CN				235, 250
D	organic acids	KINETEX–C18 column (4.6×150 mm, 5 μm)	A: 10 mM KH_2_PO_4_/H_3_PO_4_, pH=2.8;	0.6	13+2 (CT)	No	214
			B: CH_3_CN				
E	vitamins	KINETEX–C18 column (4.6×150 mm, 5 μm)	A: 5 mM C_16_H_33_N(CH_3_)_3_Br/50 mM KH_2_PO_4_, pH=2.5;	0.9	10+5 (CT)	No	261, 348
			B: CH_3_OH				

**Table 3:** Identification standard codes, standard t_R_, calibration curve equations, *R^2^*, calibration curve ranges, LOD, and LOQ of the used chromatographic methods for each calibration standard [10].

Class	Standard	Identification code	Retention time (t_R_) (min)	Wavelenght (nm)	Method	Calibration curve equation	*R^2^*	Calibration curve range (mg L^−1^)	LOD (mg L^−1^)	LOQ (mg L^−1^)
Cinnamic acids	caffeic acid	1	4.54	330	A	y = 59.046x + 200.6	0.996	111–500	0.305	1.016
	chlorogenic acid	2	3.89	330	A	y = 13.583x + 760.05	0.984	111–500	0.940	3.134
	coumaric acid	3	6.74	330	A	y = 8.9342x + 217.4	0.997	111–500	2.907	9.690
	ferulic acid	4	7.99	330	A	y = 3.3963x − 4.9524	1.000	111–500	1.245	4.150
Flavonols	hyperoside	5	10.89	330	A	y = 7.1322x − 4.583	0.999	111–500	3.372	11.241
	isoquercitrin	6	11.24	330	A	y = 8.3078x + 26.621	0.999	111–500	0.252	0.840
	quercetin	7	17.67	330	A	y = 3.4095x − 98.307	0.998	111–500	4.055	13.518
	quercitrin	8	13.28	330	A	y = 2.7413x + 5.6367	0.998	111–500	5.456	18.187
	rutin	9	12.95	330	A	y = 6.5808x + 30.831	0.999	111–500	2.937	9.790
Benzoic acids	ellagic acid	10	18.65	280	B	y = 29.954x + 184.52	0.998	62.5–250	0.611	2.035
	gallic acid	11	4.26	280	B	y = 44.996x + 261.86	0.999	62.5–250	0.435	1.451
Catechins	catechin	12	10.31	280	B	y = 8.9197x + 66.952	1.000	62.5–250	2.343	7.809
	epicatechin	13	14.30	280	B	y = 12.88x − 43.816	0.999	62.5–250	0.763	2.543
Monoterpenes	limonene	14	3.35	250	C	y = 0.1894x − 5.420	0.999	125–1000	8.654	28.847
	phellandrene	15	3.57	210	C	y = 8.783x − 145.3	0.998	125–1000	0.562	1.874
	sabinene	16	3.45	220	C	y = 18.14x − 1004	0.998	125–1000	0.094	0.314
	γ-terpinene	17	3.28	235	C	y = 0.4886x − 23.02	0.999	125–1000	17.577	58.590
	terpinolene	18	4.83	220	C	y = 26.52x + 876.8	0.999	125–1000	0.241	0.804
Organic acids	citric acid	19	5.30	214	D	y = 1.0603x − 22.092	1.000	167–1000	18.805	62.682
	malic acid	20	4.03	214	D	y = 1.415x − 80.254	0.996	167–1000	15.721	52.404
	oxalic acid	21	7.85	214	D	y = 6.4502x + 6.1503	0.998	167–1000	0.550	1.835
	quinic acid	22	3.21	214	D	y = 0.8087x − 38.021	0.998	167–1000	26.106	87.021
	succinic acid	23	3.46	214	D	y = 0.9236x − 8.0823	0.995	167–1000	7.135	23.783
	tartaric acid	24	5.69	214	D	y = 1.8427x + 15.796	1.000	167–1000	8.520	28.401
Vitamins	ascorbic acid	25	4.14	261	E	y = 42.71x + 27.969	0.999	100–1000	0.836	2.786
	dehydroascorbic acid	26	3.41	348	E	y = 4.1628x + 140.01	0.999	30–300	1.095	3.649

**Figure 7:**
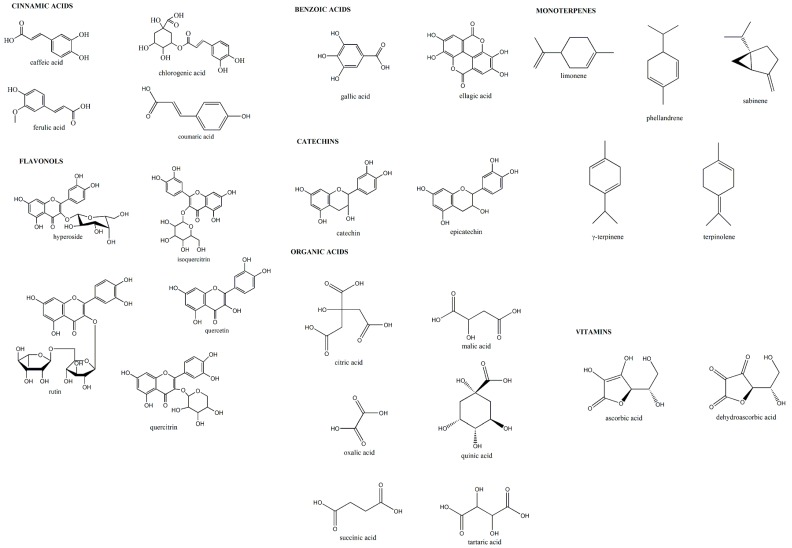
Chemical structures of the main selected biomarkers.

According to “multi-marker approach” [47], total bioactive compound content (TBCC) was determined as the sum of the most important classes of bioactive compounds present in the samples. Bioactive markers were selected comparing bud-preparation health-promoting properties and the most important compounds in literature with an important role in the positive effects on human organism (Figure 7). Four polyphenolic classes were considered: benzoic acids (ellagic and gallic acids), catechins (catechin and epicatechin), cinnamic acids (caffeic, chlorogenic, coumaric, and ferulic acids), and flavonols (hyperoside, isoquercitrin, quercetin, quercitrin, and rutin). Monoterpenes (limonene, phellandrene, sabinene, γ-terpinene, terpinolene), organic acids (citric, malic, oxalic, quinic, succinic, and tartaric acids) and vitamin C (ascorbic and dehydroascorbic acids) were also considered to obtain a complete analytical fingerprint. All results were expressed as mg per 100 g of fresh weight (FW).

### 3.6. Statistical Analysis

Results were subjected to analysis of variance (ANOVA) test for mean comparison (IBM SPSS Statistics, v. 22.0, New York, NY, USA) and HSD Tukey multiple range test (*p* < 0.05). 

## 4. Conclusions

In this study, *Ribes* and *Rubus* spp. were identified as new sources of natural antioxidants and other health-promoting compounds for use in herbal products. In particular, the results demonstrated that these bud-preparations represent a rich source of polyphenolic (catechins and flavonols) and terpenic compounds and indicated that secondary plant metabolite concentration in bud preparations is highly dependent on harvesting time and plant genotype. For this reason, the concentrations of main bioactive compounds in buds, and consequently in bud-preparations, can be opportunely defined on the basis of chemical-pharmaceutical, agricultural and environmental knowledge. The differences in the phytocomplex chemical composition of blackcurrant and blackberry justify the different medical uses of these preparations; in blackcurrant bud-extracts the most important class was organic acids (50.98%) followed by monoterpenes (14.05%), while in blackberry preparations the main bioactive classes were catechins (50.06%) and organic acids (27.34%).

The HPLC methods used in this study are simple, sensitive and reliable, and could be used for the quality evaluation and control of bud-extracts and natural medicines. The results of this research show that the assessment of chemical composition of the plant-derived products could help in find out new sources of natural antioxidants and other health-promoting compounds which could be used as natural medicines, food additives, functional foods and botanical ingredients in order to develop a new generation of standardized and effect-optimized preparations with high values of quality and safety. 

Chemical, genetic and environmental knowledge could be a useful tool for obtaining label certifications for the valorization of specific genotypes, with high clinical and pharmaceutical value: chromatographic fingerprinting could be an effective tool for herbal product characterization and authentication, natural preparation quality control (against contamination and adulteration), bioactivity evaluation of bud preparations, and standardization of all the supply chain steps.

## References

[1] Ramawat K., Dass S., Mathur M. (2009). The chemical diversity of bioactive molecules and therapeutic potential of medicinal plants. Herbal Drugs: Ethnomedicine to Modern Medicine.

[2] Verpoorte R. (2009). Medicinal plants: A renewable resource for novel leads and drugs. Herbal Drugs: Ethnomedicine to Modern Medicine.

[3] Pal S.K., Shukla Y. (2003). Herbal medicine: Current status and the future. Asian Pacific J. Cancer Prev..

[4] Chen L., Xin X.L., Yuan Q.P., Su D.H., Liu W. (2014). Phytochemical properties and antioxidant capacities of various colored berries. J. Sci. Food Agric..

[5] Donno D., Beccaro G.L., Mellano M.G., Cerutti A.K., Bounous G. (2013). Medicinal plants, chemical composition and quality: May blackcurrant buds and blackberry sprouts be a new polyphenol source for herbal preparations?. J. Appl. Bot. Food Qual..

[6] Gopalan A., Reuben S.C., Ahmed S., Darvesh A.S., Hohmann J., Bishayee A. (2012). The health benefits of blackcurrants. Food Funct..

[7] Tabart J., Franck T., Kevers C., Pincemail J., Serteyn D., Defraigne J.-O., Dommes J. (2012). Antioxidant and anti-inflammatory activities of ribes nigrum extracts. Food Chem..

[8] Dall’Acqua S., Cervellati R., Loi M.C., Innocenti G. (2008). Evaluation of *in vitro* antioxidant properties of some traditional sardinian medicinal plants: Investigation of the high antioxidant capacity of rubus ulmifolius. Food Chem..

[9] Panizzi L., Caponi C., Catalano S., Cioni P.L., Morelli I. (2002). In vitro antimicrobial activity of extracts and isolated constituents of rubus ulmifolius. J. Ethnopharmacol..

[10] Donno D., Boggia R., Zunin P., Cerutti A.K., Guido M., Mellano M.G., Prgomet Z., Beccaro G.L. (2015). Phytochemical fingerprint and chemometrics for natural food preparation pattern recognition: An innovative technique in food supplement quality control. J. Food Sci. Technol..

[11] Balunas M.J., Kinghorn A.D. (2005). Drug discovery from medicinal plants. Life Sci..

[12] Konik E.A., Jungling R.C., Bauer B.A. (2011). Herbs and dietary supplements in the european union: A review of the regulations with special focus on germany and poland. J. Diet. Suppl..

[13] Silano V., Coppens P., Larranaga-Guetaria A., Minghetti P., Roth-Ehrang R. (2011). Regulations applicable to plant food supplements and related products in the european union. Food Funct..

[14] Fürst R., Zündorf I. (2015). Evidence-based phytotherapy in europe: Where do we stand?. Planta Med..

[15] Leonti M., Casu L. (2013). Traditional medicines and globalization: Current and future perspectives in ethnopharmacology. Front. Pharmacol..

[16] Donno D., Beccaro G.L., Cerutti A.K., Mellano M.G., Bounous G., Rao A.V., Rao L.G. (2015). Bud extracts as new phytochemical source for herbal preparations: Quality control and standardization by analytical fingerprint. Phytochemicals—Isolation, Characterisation and Role in Human Health.

[17] Fong H.H.S. (2002). Integration of herbal medicine into modern medical practices: Issues and prospects. Integr. Cancer Ther..

[18] Gulati O.P., Berry Ottaway P. (2006). Legislation relating to nutraceuticals in the european union with a particular focus on botanical-sourced products. Toxicology.

[19] Knoss W., Chinou I. (2012). Regulation of medicinal plants for public health—european community monographs on herbal substances. Planta Med..

[20] Donno D., Beccaro G.L., Mellano M.G., Bonvegna L., Bounous G. (2014). Castanea spp. Buds as a phytochemical source for herbal preparations: Botanical fingerprint for nutraceutical identification and functional food standardisation. J. Sci. Food Agric..

[21] Calixto J.B. (2000). Efficacy, safety, quality control, marketing and regulatory guidelines for herbal medicines (phytotherapeutic agents). Braz. J. Med. Biol. Res..

[22] Donno D., Beccaro G.L., Mellano G.M., Cerutti A.K., Canterino S., Bounous G. (2012). Effect of agronomic and environmental conditions on chemical composition of tree-species buds used for herbal preparations. Vegetos Int. J Plant Res..

[23] Vagiri M., Ekholm A., Öberg E., Johansson E., Andersson S.C., Rumpunen K. (2013). Phenols and ascorbic acid in black currants (ribes nigrum l.): Variation due to genotype, location, and year. J. Agric. Food Chem..

[24] Liang Y.-Z., Xie P., Chan K. (2004). Quality control of herbal medicines. J. Chromatogr. B.

[25] Júnior J.O.C.S., Costa R.M.R., Teixeira F.M., Barbosa W.L.R. (2011). Processing and quality control of herbal drugs and their derivatives. Quality Control of Herbal Medicines and Related Areas.

[26] Rossi Forim M., Perlatti B., Soares Costa E., Facchini Magnani B., Donizetti de Souza G. (2015). Concerns and considerations about the quality control of natural products using chromatographic methods. Curr. Chromatogr..

[27] Zhang Y., Sun S., Dai J., Wang W., Cao H., Wu J., Gou X. (2011). Quality control method for herbal medicine-chemical fingerprint analysis. Quality Control Herbal Medicines and Related Areas.

[28] He X.G. (2000). On-line identification of phytochemical constituents in botanical extracts by combined high-performance liquid chromatographic-diode array detection-mass spectrometric techniques. J. Chromatogr. A.

[29] Shi Z.-Q., Song D.-F., Li R.-Q., Yang H., Qi L.-W., Xin G.-Z., Wang D.-Q., Song H.-P., Chen J., Hao H. (2014). Identification of effective combinatorial markers for quality standardization of herbal medicines. J. Chromatogr. A.

[30] Ong E.S. (2004). Extraction methods and chemical standardization of botanicals and herbal preparations. J. Chromatogr. B.

[31] Faller A.L.K., Fialho E. (2010). Polyphenol content and antioxidant capacity in organic and conventional plant foods. J. Food Compos. Anal..

[32] Scalbert A., Johnson I.T., Saltmarsh M. (2005). Polyphenols: Antioxidants and beyond. Am. J. Clin. Nutr..

[33] Hodges D.M., Kalt W. Health Functionality of Small Fruit. http://www.actahort.org/books/626/626_1.htm.

[34] Shukitt-Hale B., Lau F.C., Joseph J.A. (2008). Berry fruit supplementation and the aging brain. J. Agric. Food Chem..

[35] Dvaranauskaitė A., Venskutonis P.R., Raynaud C., Talou T., Viškelis P., Sasnauskas A. (2009). Variations in the essential oil composition in buds of six blackcurrant (*Ribes nigrum* L.) cultivars at various development phases. Food Chem..

[36] Kerslake M.F., Menary R.C. (1985). Varietal differences of extracts from blackcurrant buds (*Ribes nigrum* L.). J. Sci. Food Agric..

[37] Dabbou S., Sifi S., Rjiba I., Esposto S., Taticchi A., Servili M., Montedoro G.F., Hammami M. (2010). Effect of pedoclimatic conditions on the chemical composition of the sigoise olive cultivar. Chem. Biodivers..

[38] Donno D., Beccaro G.L., Mellano M.G., Cerutti A.K., Marconi V., Bounous G. (2013). Botanicals in ribes nigrum bud-preparations: An analytical fingerprinting to evaluate the bioactive contribution to total phytocomplex. Pharm. Biol..

[39] Gong F., Wang B.-T., Liang Y.-Z., Chau F.-T., Fung Y.-S. (2006). Variable selection for discriminating herbal medicines with chromatographic fingerprints. Anal. Chim. Acta.

[40] Bian Q., Yang H., Chan C.O., Jin D., Mok D.K., Chen S. (2013). Fingerprint analysis and simultaneous determination of phenolic compounds in extracts of curculiginis rhizoma by hplc-diode array detector. Chem. Pharm. Bull..

[41] Feng X., Kong W., Wei J., Ou-Yang Z., Yang M. (2014). Hplc fingerprint analysis combined with chemometrics for pattern recognition of ginger. Pharm. Biol..

[42] Lugasi A., Hóvári J., Kádár G., Dénes F. (2011). Phenolics in raspberry, blackberry and currant cultivars grown in hungary. Acta Aliment..

[43] Dugo P., Cacciola F., Donato P., Jacques R.A., Caramao E.B., Mondello L. (2009). High efficiency liquid chromatography techniques coupled to mass spectrometry for the characterization of mate extracts. J. Chromatogr. A.

[44] Kesting J.R., Huang J., Sorensen D. (2010). Identification of adulterants in a chinese herbal medicine by lc-hrms and lc-ms-spe/nmr and comparative *in vivo* study with standards in a hypertensive rat model. J. Pharm. Biomed. Anal..

[45] Liang Q.L., Qu J., Luo G.A., Wang Y.M. (2006). Rapid and reliable determination of illegal adulterant in herbal medicines and dietary supplements by lc/ms/ms. J. Pharm. Biomed. Anal..

[46] Ordre_National_des_Pharmaciens (1965). Pharmacopée française, codex medicamentarius gallicus, codex français: Monographie, préparations homéopathiques.

[47] Mok D.K.W., Chau F.T. (2006). Chemical information of chinese medicines: A challenge to chemist. Chemomet. Intell. Lab. Syst..

